# Nitrogen and Phosphorus Interplay in Lupin Root Nodules and Cluster Roots

**DOI:** 10.3389/fpls.2021.644218

**Published:** 2021-03-03

**Authors:** José J. Pueyo, Miguel A. Quiñones, Teodoro Coba de la Peña, Elena E. Fedorova, M. Mercedes Lucas

**Affiliations:** ^1^Institute of Agricultural Sciences, ICA-CSIC, Madrid, Spain; ^2^Centro de Estudios Avanzados en Zonas Áridas (CEAZA), La Serena, Chile; ^3^K.A. Timiryazev Institute of Plant Physiology, Russian Academy of Science, Moscow, Russia

**Keywords:** lupin, *Lupinus*, phosphorus, nitrogen, cluster roots, nodules, nutrient deficiency, nutrient interactions

## Abstract

Nitrogen (N) and phosphorus (P) are two major plant nutrients, and their deficiencies often limit plant growth and crop yield. The uptakes of N or P affect each other, and consequently, understanding N–P interactions is fundamental. Their signaling mechanisms have been studied mostly separately, and integrating N–P interactive regulation is becoming the aim of some recent works. Lupins are singular plants, as, under N and P deficiencies, they are capable to develop new organs, the N_2_-fixing symbiotic nodules, and some species can also transform their root architecture to form cluster roots, hundreds of short rootlets that alter their metabolism to induce a high-affinity P transport system and enhance synthesis and secretion of organic acids, flavonoids, proteases, acid phosphatases, and proton efflux. These modifications lead to mobilization in the soil of, otherwise unavailable, P. White lupin (*Lupinus albus*) represents a model plant to study cluster roots and for understanding plant acclimation to nutrient deficiency. It tolerates simultaneous P and N deficiencies and also enhances uptake of additional nutrients. Here, we present the structural and functional modifications that occur in conditions of P and N deficiencies and lead to the organogenesis and altered metabolism of nodules and cluster roots. Some known N and P signaling mechanisms include different factors, including phytohormones and miRNAs. The combination of the individual N and P mechanisms uncovers interactive regulation pathways that concur in nodules and cluster roots. *L. albus* interlinks N and P recycling processes both in the plant itself and in nature.

## Introduction

Nitrogen (N) and phosphorus (P) are the two main nutrients used by plants and, together with potassium (K), the most extensively used fertilizer elements that drive plant growth and crop yield. P and N deficiencies often limit primary productivity in both agricultural and natural systems (Menge et al., 2012; Ågren et al., 2012). The importance of N–P interactions has long been recognized; however, the metabolism and signaling pathways of N and P have been quite often studied separately. Some recent works are reporting progress in understanding N–P interactions, and the mechanisms integrating N–P interactive regulation pathways are being uncovered (Ueda and Yanagisawa, 2019; Hu and Chu, 2020). The uptakes of N or P affect each other, suggesting strategies to maintain N–P nutritional balance in plants (Güsewell, 2004).

Most studies on N–P interactions have been performed in model or in important crop plants, including N_2_-fixing legumes. The genus *Lupinus* is a singular one among legumes. Lupins can fix atmospheric N_2_ as a result of the symbiotic interaction with rhizobia. While it is generally accepted that lupins do not form mycorrhizas (Lambers et al., 2013), some lupin species are able to respond to P deficiency by developing a structural and functional root modification, the cluster roots. Cluster roots are mostly a characteristic of the Proteaceae but also of a few other plant species (Watt and Evans, 1999). They are associated with intense mobilization of P and other nutrients by root-induced chemical changes in the rhizosphere. They are bottlebrush-like clusters of hundreds of rootlets with limited growth that arise from the pericycle opposite the protoxylem poles mostly along the lateral roots (Neumann and Martinoia, 2002).

Lupin is a crop with great potential to be further developed for high-protein production (Lucas et al., 2015), as alternative protein sources are increasingly becoming a necessity (De Ron et al., 2017), and also for soil phytoremediation and recovery of degraded soils (Fernández-Pascual et al., 2007; Quiñones et al., 2013). The microsymbionts associated with lupins, which belong primarily to the genus *Bradyrhizobium*, penetrate the root at the junction between the root hair base and an adjacent epidermal cell. Bacteria invade the subepidermal cortical cell underneath the root hair, and the infected cell divides repeatedly to form the infected zone of the young nodule (González-Sama et al., 2004). Inside the infected cells, rhizobia are surrounded by a membrane to form the symbiosomes, and bacteria differentiate to N_2_-fixing bacteroids. Symbiosomes are distributed between the daughter cells in an analogous fashion to other cell organelles (Fedorova et al., 2007; Coba de la Peña et al., 2018). Endoreduplication processes associated with nodule development take place in a similar manner to other legumes (González-Sama et al., 2006).

Most *Lupinus* species (close to 300) are native of the New World (America), with only a small number of representatives in the Old World (Mediterranean Basin and Eastern Africa) (Aïnouche and Bayer, 1999). Cluster roots were initially thought to be confined to a few Old World species (Skene, 2000). Later studies have shown that some species in both the Old and the New World are capable of forming cluster roots, including the most cultivated lupin species worldwide, *L. albus*, *L. angustifolius*, and *L. luteus*, originally from the Old World, and the New World species *L. mutabilis* (Table 1). Some species need very low P concentrations to form cluster roots (Hocking and Jeffery, 2004).

**TABLE 1:** *Lupinus* species that have been tested for cluster roots formation.

	Cluster roots	References
**Old World species^1^**		
*L. albus*	Yes	Gardner et al., 1982
*L. anatolicus*	n.t.	
*L. angustifolius*	Yes/no	Egle et al., 2003; Hocking and Jeffery, 2004
*L. atlanticus*	Yes	Clements et al., 1993; Abdolzadeh et al., 2010
*L. consentinii*	Yes	Trinick, 1977
*L. digitatus*	Yes	Clements et al., 1993
*L. hispanicus*	Yes	Hocking and Jeffery, 2004
*L. luteus*	Yes	Hocking and Jeffery, 2004
*L. mariae-josephi*	n.t.	
*L. micranthus*	Yes	Clements et al., 1993; Abdolzadeh et al., 2010
*L. palaestinus*	Yes	Clements et al., 1993
*L. pilosus*	Yes	Clements et al., 1993
*L. princei*	Yes	Clements et al., 1993
*L. somaliensis*	Extinct	
**New World species^2^**		
*L. arboreus*	No	Skene and James, 2000
*L. guadalupensis*	No	Lambers et al., 2013
*L. lepidus*	Yes*	Lambers et al., 2012
*L. mutabilis*	Yes/no	Hocking and Jeffery, 2004; Pearse et al., 2006
*L. polyphyllus*	Yes	Razavi et al., 2017
*L. sericeus*	cho	Lambers et al., 2013
*L. subcarinosus*	No	Lambers et al., 2013
*L. sulphureus*	No	Lambers et al., 2013
*L. texensis*	No	Lambers et al., 2013

*^1^All known Old World Lupinus species are listed. ^2^Only tested New World species are included. n.t. indicates not tested; Yes^∗^, “cluster-like” root formation; cho, enhanced carboxylate release by roots; Yes/no, discrepancies between different studies.*

Unlike N, P is a non-renewable resource, and many recent studies anticipate that readily available sources of P will be eventually depleted in 200–300 years, or even by the end of this century (Valentine et al., 2017; Alewell et al., 2020). In this context, lupin appears as a perfect crop, as it reduces the need for P (and N) fertilizers, contributing to sustainable agriculture practices, and it is suitable for impoverished soils with important amounts of P which is hardly available for most plants. They can thrive in these poor soils and become productive crops or help restore degraded landscapes (Coba de la Peña and Pueyo, 2012).

There is a need to better understand the distinct N–P interactions that take place in lupins. Here, we will review the still limited knowledge of the interplay between N_2_-fixing nodules and cluster roots in lupins. Most published studies are on white lupin (*L. albus*), which is considered a model plant to study cluster roots and for understanding plant acclimation to nutrient deficiency (Hufnagel et al., 2020). The vast majority of published works deal with only one organ, either nodules or cluster roots, mostly under controlled conditions in different hydroponic systems. We will review the existing literature and combine studies on one organ or the other to better understand the connections between their metabolisms and highlight the importance of the N–P interactive regulation pathways taking place in both organs, which, functioning together, are able to interlink the N and P recycling processes and can reduce or even suppress the need for fertilization of this crop.

## P and N Deficiencies Affect the Formation and Metabolism of Lupin Cluster Roots and Root Nodules

White lupin high tolerance to P deficiency is mediated by the formation of cluster roots, which may also be stimulated by iron deficiency (Lamont, 2003; McCluskey et al., 2004). Metabolic alterations occur that include an enhanced synthesis and secretion of organic acids, the induction of a high-affinity P transport system (Liu et al., 2001), secreted acid phosphatases, proton efflux (Vance et al., 2003), and flavonoid secretion (Tomasi et al., 2008). In *L. albus*, citrate production represents an important percentage of the plant dry weight (Dinkelaker et al., 1989), and it increases extraordinarily in the absence of external supplies of P (Johnson et al., 1996). This increase takes place by blocking citrate catabolism in the cytosol and increasing the supply of pyruvate and oxaloacetate (Kihara et al., 2003). Citrate is released through permeable channels (Zhang et al., 2004), coupled with H^+^-ATPases (Tomasi et al., 2009). Recently, an aluminum-independent specific malate transporter has been identified, which is upregulated under P deficit and can also transport metals from cluster roots to shoots and most likely to nodules via xylem (Zhou et al., 2019). Organic anion secretion and rhizosphere acidification mobilize not only P but also other nutrients like Fe, Ca, Mn, or Zn, increasing their uptake rates (Lamont, 2003). P is loaded into the xylem through transporters (Lucena et al., 2019).

Phosphorus deficiency initially reduces the levels of sugars (fructose, glucose, and sucrose) in shoots, and phosphorylated metabolites such as glycerol-3-phosphate, fructose-6-phosphate, glucose-6-phosphate, or myo-inositol-phosphate levels decrease in both shoots and roots of *L. albus* (Müller et al., 2015). Prolonged P deficiency eliminates such effects on sugar metabolite levels, but the level of phosphorylated metabolites is reduced. Organic acids, amino acids, and several shikimate pathway product levels are increased in P-deficient roots and shoots, suggesting that white lupins adjust their carbohydrate partitioning between root and shoot to supply their developing root system as an early response to P deficit (Müller et al., 2015).

The plant enzymes phosphoenolpyruvate carboxylase (PEPC) and malate dehydrogenase (MDH) play a crucial role in carbon metabolism of root nodules and cluster roots. In nodules, they provide carbon in the form of organic acids for bacteroid functioning (Rosendahl et al., 1990). In cluster roots, expression of MDH and PEPC is highly increased and a part of the exuded organic acids come from CO_2_ fixed by PEPC (Johnson et al., 1996; Uhde-Stone et al., 2003). About 30% of the organic C released is derived from CO_2_ fixation via PEPC in *L. albus* P-deficient roots (Johnson et al., 1996).

The formation of cluster roots in lupin induces important changes in gene expression (Rath et al., 2010). Transcriptomic analyses show that white lupin adaptation to P deficiency induces transcription of genes encoding proteins related to P transport, signaling proteins, transcription factors, genes involved in glycolysis and the alternative respiratory pathway, genes involved in malate biosynthesis, and several others (O’Rourke et al., 2013; Wang et al., 2014; Venuti et al., 2019). The genetic, structural, and metabolic changes described above that lead to the formation and functioning of cluster roots take place when N is not a limiting factor. When N is also deficient, additional changes occur in lupin roots.

The first and most evident effect of N deficiency on legumes is the formation of symbiotic nodules, the organs where nitrogen fixation takes place. Nodule organogenesis has been extensively studied (Brewin, 1991; Schultze and Kondorosi, 1998), so we will not go into depth in the subject here. Basically, nodulation starts with a dialogue between the plant and the bacteria involving plant-secreted flavonoids and rhizobial Nod factors. Bacteria enter the root by infection of a root hair (infection thread), or intercellularly (Ibáez et al., 2017). Depending on their origin and development, nodules can be determinate (round with limited growth) or indeterminate (cylindrical with an active apical meristem) (Brewin, 1991). Lupins present an unusual way of intercellular infection and a characteristic indeterminate “lupinoid” nodule with a lateral meristem that allows growth surrounding the lupin root (González-Sama et al., 2004). In general, temperate legume nodules, including lupin, export amino acids, Gln and Asn, while tropical legume nodules export ureides. Nodulation of white lupin is inhibited by N, and the inhibitory effects depend on the N source (Guo et al., 1992). The inhibition mechanisms are complex and involve miRNAs and transcription factors (Lin et al., 2018; Xu et al., 2020). Transcriptomic analyses have revealed numerous genes expressed and regulated during nodule development, transport, and metabolism (Udvardi and Poole, 2013 and references therein). The amino acids synthesized in the nodule are transported symplasmically to the xylem or released through transporters to the root apoplasm. Transfer to the phloem can be symplasmic or apoplasmic, depending on plasmodesmata and substrate concentrations. Exporters in the plasma membranes of the phloem parenchyma would be required for apoplasmic transport (Tegeder, 2014 and references therein). An amino acid permease associated with transport from the nodule has been identified in pea, and it is upregulated under N deficit (Garneau et al., 2018).

Low levels of N enhance formation of cluster roots under P deficiency, while high N levels have an inhibitory effect (Dinkelaker et al., 1995). The form of nitrogen acquisition by the plant has an effect on cluster roots (Sas et al., 2002). N_2_-fixing white lupin plants were compared with plants supplied with (NH_4_)_2_SO_4_ and NH_4_NO_3_. In the latter, N concentration was higher, and there was greater H^+^ extrusion and higher cluster root formation. The investment of resources in nodule organogenesis and functioning might decrease their allocation to cluster root formation (Sas et al., 2002). However, nodules are P sinks and it would be logical that cluster root number increased when the plant depends on symbiotic N. Wang et al. (2019a) also compared nodulated *L. albus* plants with plants that were provided with an amount of nitrate that was the same as the amount acquired by the nodulated plants, from both air and nutrient solution. They concluded that nodulation promoted cluster root formation in *L. albus* under low P.

*Hakea actites* (Proteaceae) cluster roots are capable of using glycine as N source. The amino acid is then metabolized via an aminotransferase to serine and other amino compounds (Schmidt and Stewart, 1999). Moreover, enhanced peptidase activity and high expression of amino acid and peptide transporters have been observed in *Hakea* cluster roots when protein is the N source (Schmidt et al., 2003; Paungfoo-Lonhienne et al., 2009). In fact, secretion by *Hakea* cluster roots of proteases capable of hydrolyzing proteins in the soil has also been reported; amino acids and di- and tri-peptides enter the root cell via plasma membrane transporters. The possibility of whole protein uptake has been suggested (Paungfoo-Lonhienne et al., 2008, 2009). *L. albus* cluster roots are also able to take up glycine (Hawkins et al., 2005), and when cattle manure is used as fertilizer, N accumulation in lupin is higher than the decrease in soil inorganic N indicating that lupin is capable of acquiring N from organic N sources (Watanabe et al., 2006). Whereas microbes in the rhizosphere can also secrete proteolytic enzymes, a recent study suggests that lupin cluster roots, rather than rhizosphere microorganisms, are responsible for the degradation of organic N (Fujiishi et al., 2020).

In general, relatively large amounts of P are required for N_2_ fixation (Vitousek et al., 2010; Valentine et al., 2017). P is preferentially allocated to nodules to maintain symbiotic N_2_ fixation during P deficiency, which might result in reduced plant growth (Høgh-Jensen et al., 2002). Nodules are strong P sinks, and bacteroids are able to scavenge P from the host cells to sustain their metabolism (Al-Niemi et al., 1997, 1998; Valentine et al., 2011). P can be absorbed from the soil by the nodule surface or translocated from the roots to the nodule via the vascular tissue (Al-Niemi et al., 1998). Most of the mobilized nutrients are transported to the nodule via xylem or phloem-transported from cluster roots. The high rate of RNA synthesis linked to the turnover of oxygen-damaged nitrogenase requires significant amounts of P. This is the main reason why nodules have a much higher demand for P compared to other plant tissues (Raven, 2012). Physiological studies have shown that, in the nodule, bacteroids accumulate high levels of P required to sustain nitrogenase activity (Sulieman and Tran, 2015).

Transcriptomics and metabolomics have been used to examine the effect of P deficiency on nodule functioning in several legumes (Abdelrahman et al., 2018 and references therein). P deficiency has an effect on legume nodule functioning, and numerous genes have been identified that are involved in nodule response to P limitations, some of which coincide with those induced in cluster roots, such as those related to glycolysis and the alternative respiratory pathway in bean nodules (Hernández et al., 2009). In *Medicago truncatula*, P deficiency induces downregulation of leghemoglobins and other nodule related genes; moreover, P deficiency and addition of nitrate have similar effects on the transcriptome of *M. truncatula* nodules (Liese et al., 2017). When it comes to white lupin nodules, P deficiency has not any remarkable effect on their functioning, when cluster roots are efficient in P mobilization and uptake (Schulze et al., 2006; Thuynsma et al., 2014a). White lupin plants grown under low P and low N that formed functional cluster roots and root nodules were used to examine the effects of N deficit in conditions of sufficient P supply on both organs. In cluster roots, there was a decrease in enzyme activities PEPC, pyruvate kinase, malic enzyme, and MDH, which are related to CO_2_ fixation and organic acid synthesis. The opposite effect was observed in nodules, probably to compensate the organic acids provided by the cluster root cells under P deficiency (Thuynsma et al., 2014b). High P inhibits cluster root formation in white lupin, and it has been proposed that citrate exudation is regulated by shoot P status, while proton efflux depends on local P supply (Li et al., 2008).

## N–P Interactive Regulation Pathways

In white lupin roots, secreted acid phosphatase and phosphate transporter genes show significant induction in response to P deficiency (Liu et al., 2005). In addition, both these genes are expressed in N_2_-fixing nodules, indicating a connection between P deficiency and factors related to N_2_ fixation and metabolism. Sugars and/or sugar metabolites play an important role in signal transduction during N assimilation and are required for efficient N_2_ fixation, as they are also crucial for P-deficiency signal transduction (Liu et al., 2005).

Enhanced nodulation in cluster root zones has been reported (Schulze et al., 2006). The increased number of nodules might be a result of increased auxin concentrations in cluster root zones. Auxins are involved in cluster root initiation (Gilbert et al., 2000; Gallardo et al., 2019). The location of the nodules in the proximity of cluster roots under P deficiency suggests that tissue P concentration is involved in the regulation of nodule initiation (Almeida et al., 2000). Nodule formation requires high auxin levels to initiate cell division and the formation of the nodule primordium. Not only is auxin transported to the nodule, but also it might be synthesized *in situ* (Fedorova et al., 2005). *YUCCA* genes involved in auxin biosynthesis are expressed in nodule primordia (Wang et al., 2019b). In cluster roots, the expression of these genes has also been reported (Secco et al., 2014; Wang et al., 2015).

Cytokinin appears to be involved in the systemic suppression of P signaling (Martín et al., 2000). Several genes involved in cytokinin degradation have been reported in cluster roots (O’Rourke et al., 2013). P deprivation inhibits the action of cytokinin by reducing its concentration and decreasing the expression of cytokinin receptors in *Arabidopsis* (Franco-Zorrilla et al., 2005). Cytokinins play also a major role in the regulation of nodule formation and functioning. A homolog of cytokinin receptor AtAHK3 has been related to nodule organogenesis in *L. albus* (Coba de la Peña et al., 2008).

Ethylene also plays a pivotal role in modulating both local and systemic responses to P deficit. A decrease in extracellular P results in enhanced ethylene biosynthesis and responsiveness in roots, which promotes the necessary changes in the root system architecture to improve P mobilization capability (Nagarajan and Smith, 2012). Ethylene production in cluster roots might have a negative effect on nodule formation, but in functioning nodules, an ACC deaminase gene, which encodes an enzyme that catalyzes the degradation of the ethylene precursor ACC into ammonium and α-ketobutyrate to reduce ethylene levels, is expressed in bacteroids (Nascimento et al., 2018).

Plant microRNAs affect both the N-signaling pathways (Fischer et al., 2013) and the P-signaling as well. Expression of miR399 is upregulated under Pi deficiency in white lupin, suggesting a possible role as a long-distance signal of P deficiency (Zhu et al., 2010). Recently, miR399 has also been linked to legume nodule functional processes (Figueredo et al., 2020). The importance of ethylene and miR399 has been reported in the regulation of responses to Fe and P deficiencies (Lucena et al., 2019). Nitric oxide (NO) is another signaling molecule related to both N and P signaling. In *L. albus*, P deficiency greatly enhances NO production in cluster roots (Wang et al., 2010) and NO synthase activity has been reported in white lupin nodules (Cueto et al., 1996).

Altogether, it appears that P- and N-signaling pathways are interconnected by hormones and miRNAs, related to cluster root and nodule formation, functioning, and communication with each other and with the aboveground part of the plant. Both cluster roots and root nodules require resources, in the form of P, N, and C for growth and functionality. When any of them is limited, competition might occur between organs (Lynch and Ho, 2005; Kleinert et al., 2014). In *L. albus*, there are significant differences in specialized belowground allocation to cluster roots and nodules. Cluster roots improve the P nutrition to the nodules (Schulze et al., 2006; Mortimer et al., 2008). Valuable C and P resources are redirected from cluster roots to nodules during adequate P supply. Nodules seem to have adapted to maintain functionality and efficiency of N_2_ fixation, despite changes in P availability and costs associated with cluster roots formation and metabolism. The increase in the C costs of cluster root formation during P deficit is needed to improve the P nutrition of nodules in order to maintain N_2_ fixation under P stress. The high allocation of P to the nodules also leads to sink stimulation via photosynthesis for increased C supply (Thuynsma et al., 2014a).

## Discussion

The excessive use of N and P fertilizers has pernicious consequences, such as eutrophication of water sources. Improvement of crop nutrient acquisition is becoming crucial for both environmental and economic reasons (Vance, 2001; Welch and Graham, 2004). Lupins are a promising crop, which at present is not sufficiently exploited in agriculture. Lupins may also act as ecosystem facilitators by rendering P available for neighboring plants, as demonstrated for *L. albus* (Gardner and Boundy, 1983; Horst and Waschkies, 1987; Cu et al., 2005). Different *Lupinus* species have been used in ecosystem recovery (Lambers et al., 2012), and the benefits of using lupin in intercropping and crop rotation are also well known (Doyle et al., 1989). Lupins possess great advantages in agriculture and ecosystem restoration, but for the same reason, sometimes they might become invasive species (Jauni and Ramula, 2017).

N_2_-fixing legumes that do not form cluster roots have also evolved mechanisms to cope with P limitation. In general, nodules develop very flexible mechanisms for P recycling and internal P conservation, rather than specific mechanisms to acquire external P (Vardien et al., 2016). Reallocation of P from both leaves and roots to nodules has been described (Esfahani et al., 2016). Plants are capable of mobilizing P from internal resources, such as phospholipids and nucleic acids (Hernández et al., 2009). Additionally, roots are able to secrete some P-mobilizing compounds. However, despite these adaptations, legume nodules are sensitive to long-term P deficiency (Sa and Israel, 1991). Lupins that do not form cluster roots can be more resilient to P deficiency than other legumes (Le Roux et al., 2006; Lambers et al., 2013). *L. albus* is the most efficient species in cluster root formation and functioning. Schulze et al. (2006) evaluated white lupin nodulation and N_2_ fixation under P deficit. Plants were grown in the absence of N and subjected to sufficient or no P. Nodulation and N_2_ fixation were highly tolerant to P deficiency. There were no differences in N_2_ fixation rates between +P and −P supply, and shoots of nodulated plants did not show any signs of nutrient stress when grown under N and P deficiencies.

When lupins obtain N through symbiotic N_2_ fixation, there must be a trade-off in resource allocation between root nodules and cluster roots (Thuynsma et al., 2014a). Complex signaling systems involving different actors are elicited by N and P. These include hormones, miRNAs, and transcription factors and are dependent on N and P availability, the plant nutrients levels and their homeostasis in cluster roots and nodules. Lupin species also represent an important link that couples P and N cycles.

**FIGURE 1:**
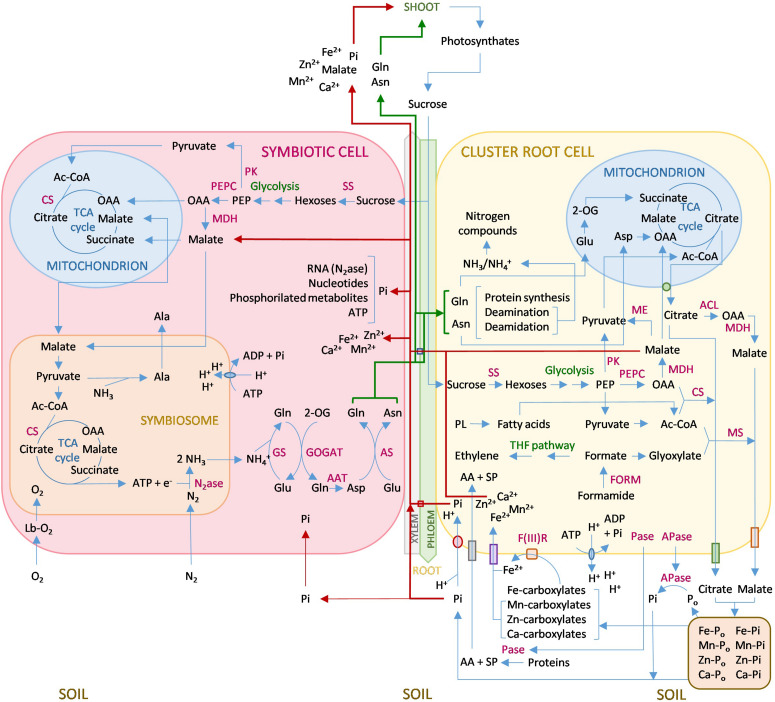
Simplified metabolism of *Lupinus albus* nodules and cluster roots under P and N deficiencies. In summary, sucrose is transported from the shoot via the phloem, and glycolysis is activated in both the symbiotic cells and the cluster root cells to increase the amount of malate and OAA, which are integrated into the TCA cycles running in both mitochondria and symbiosomes. In cluster root cells, malate is also secreted to the soil through a specific transporter and may be transported via xylem to fuel the symbiotic cell and to the aerial organs. In nodules, there is an upregulation of MDH, while in cluster roots PEPC is the principal upregulated glycolysis enzyme. In cluster roots, formate is produced by an upregulation of FORM, which leads to activation of the THF pathway, which produces ethylene. Formate is also used to synthesize malate by MS. Phospholipid degradation occurs to provide Pi and Ac-CoA, which in turn combines with OAA to yield citrate catalyzed by CS, or with glyoxylate to form malate by MS. Malate and citrate keep a balance mediated by the TCA cycles and several enzymes, inducing ACL, MDH, MS, and CS. Citrate is mainly produced in the mitochondrion and secreted to the soil through permeable channels. This process is associated with a membrane H^+^ ATPase, and protons are also released, leading to soil acidification. Additionally, APases are secreted to the soil. The combined action of carboxylates, the acidification of the medium, and the secreted APases is capable of mobilizing unavailable P in the soil and generate Pi, which is co-transported with protons into the root cell. Pi can reach the symbiotic cell by absorption through the nodule surface or, mainly, be transported via xylem. Pi is needed in the first place for the synthesis of nitrogenase RNA, which has a very high turnover. Carboxylates also chelate cations that bind to P, mainly Fe^3+^, which is reduced to Fe^2+^ by a plasma membrane Fe(III)R and enters the cell via a Fe^2+^ transporter. Other micronutrients are also mobilized and enter the root cell together with Fe^2+^. The nutrients are transported to the symbiotic cell and the rest of the plant via xylem. In the nodule, O_2_ permeates the cortex to bind leghemoglobin, controlling the right levels for nitrogenase functioning in the symbiosome, which produces NH_3_ that exits the symbiosome and is protonated in the symbiotic cell and then converted to Asn and Gln in a process catalized by several enzymes GS, GOGAT, AAT, and AS. Asn and Gln are transported to the plant, including the cluster root cells, where, among other functions, they are converted to Asp that enters the mitochondrion to fuel the TCA cycle through OAA, and to Glu that is converted to 2-OG and also enters the TCA cycle. Lupin cluster roots are also capable of secreting proteases that degrade organic N to amino acids and small di- and tri-peptides that enter the root cell through membrane transporters. Red arrows indicate the transport of Pi, malate, and nutrients to the symbiotic cell and the shoots. Green arrows indicate transport of Asn and Gln. Abbreviations: 2-OG, 2-oxoglutarate; AA, amino acids; AAT, aspartate aminotransferase; ACL, ATP citrate lyase; Ac-CoA, acetyl CoA; APase, acid phosphatase; AS, asparagine synthetase; CS, citrate synthase; F(III)R, F^3+^ reductase; FORM, formamidase; GOGAT, Glu synthase; GS, Gln synthetase; Lb, leghemoglobin; MDH, malate dehydrogenase; MS, malate synthase; N_2_ase, nitrogenase; OAA, oxaloacetate; Pase, protease; PEP, phosphoenolpyruvate; PEPC, phosphoenolpyruvate carboxylase; PL, phospholipids; PLA1, phospholipase A; PK, PEP kinase; SP, small peptides; SS, sucrose synthase; TCA, tricarboxylic acid; THF pathway, tetrahydrofolate pathway.

To summarize this review, Figure 1 shows a schematic representation of the interactions between N and P in the interrelated metabolisms of lupin nodules and cluster roots. In this scheme, we combine the effects that P and N deficiencies induce in both organs, and the P–N-regulated feedback. However, most of the information comes from studies on the effect of one nutrient on the metabolism of one organ. A comprehensive study of both organs under simultaneous P and N deficiencies and analyses of all the metabolic pathways involved is still necessary to fully comprehend the unique and complex interplay that takes place in lupin roots.

## Author Contributions

JJP conceived this review, wrote the first draft, and designed the figure and table. All authors contributed to the writing of the manuscript, proofread, and approved the final version.

## Conflict of Interest

The authors declare that the research was conducted in the absence of any commercial or financial relationships that could be construed as a potential conflict of interest.

## References

[B1] Abdelrahman M. El-Sayed M. Hashem A. Abd_Allah E. F. Alqarawi A. A. Burritt D. J. (2018). Metabolomics and transcriptomics in legumes under phosphate deficiency in relation to nitrogen fixation by root nodules. *Front. Plant Sci.* 9:922. 10.3389/fpls.2018.00922 30050543 PMC6052890

[B2] Abdolzadeh A. Wang X. Veneklaas E. J. Lambers H. (2010). Effects of phosphorus supply on growth, phosphate concentration and cluster-root formation in three *Lupinus* species. *Ann. Bot.* 105 365–374. 10.1093/aob/mcp297 20037142 PMC2826247

[B3] Ågren G. I. Wetterstedt J. ÅM. Billberger M. F. K. (2012). Nutrient limitation on terrestrial plant growth – modeling the interaction between nitrogen and phosphorus. *New Phytol.* 194 953–960. 10.1111/j.1469-8137.2012.04116.x 22458659

[B4] Aïnouche A.-K. Bayer R. J. (1999). Phylogenetic relationships in *Lupinus* (*Fabaceae*: *Papilionoideae*) based on internal transcribed spacer sequences (ITS) of nuclear ribosomal DNA. *Am. J. Bot.* 86 590–607. 10.2307/2656820 10205079

[B5] Alewell C. Ringeval B. Ballabio C. Robinson D. A. Panagos P. Borrelli P. (2020). Global phosphorus shortage will be aggravated by soil erosion. *Nat. Commun.* 11:4546. 10.1038/s41467-020-18326-7 PMC7486398 32917863

[B6] Almeida J. P. F. Hartwig U. A. Frehner M. Nösberger J. Lüscher A. (2000). Evidence that P deficiency induces N feedback regulation of symbiotic N2 fixation in white clover (*Trifolium repens* L.). *J. Exp. Bot.* 348 1289–1297. 10.1093/jexbot/51.348.1289 10937705

[B7] Al-Niemi T. S. Kahn M. L. McDermott T. R. (1997). P metabolism in the bean-*Rhizobium tropici* symbiosis. *Plant Physiol.* 113 1233–1242. 10.1104/pp.113.4.1233 12223671 PMC158246

[B8] Al-Niemi T. S. Kahn M. L. McDermott T. R. (1998). Phosphorus uptake by bean nodules. *Plant Soil* 198 71–78.

[B9] Brewin N. J. (1991). Development of the legume root nodule. *Annu. Rev. Cell Dev. Biol.* 7:191. 10.1146/annurev.cb.07.110191.001203 1809347

[B10] Clements J. C. White P. F. Buirchell B. J. (1993). The root morphology of *Lupinus angustifolius* in relation to other *Lupinus* species. *Aust. J. Agric. Res.* 44 1367–1375. 10.1071/ar9931367

[B11] Coba de la Peña T. Cárcamo C. B. Almonacid L. Zaballos A. Lucas M. M. Balemenos D. (2008). A cytokinin receptor homologue is induced during root nodule organogenesis and senescence in *Lupinus albus* L. *Plant Physiol. Biochem.* 46 219–225. 10.1016/j.plaphy.2007.10.021 18060799

[B12] Coba de la Peña T. Fedorova E. Pueyo J. J. Lucas M. M. (2018). The symbiosome: legume and rhizobia co-evolution toward a nitrogen-fixing organelle? *Front. Plant Sci.* 8:2229. 10.3389/fpls.2017.02229 29403508 PMC5786577

[B13] Coba de la Peña T. Pueyo J. J. (2012). Legumes in the reclamation of marginal soils, from cultivar and inoculant selection to transgenic approaches. *Agron. Sustain. Dev.* 32 65–91. 10.1007/s13593-011-0024-2

[B14] Cu S. T. T. Hutson J. Schuller K. A. (2005). Mixed culture of wheat (*Triticum aestivum* L.) with white lupin (*Lupinus albus* L.) improves the growth and phosphorus nutrition of the wheat. *Plant Soil* 272 143–151. 10.1007/s11104-004-4336-8

[B15] Cueto M. Hernandez-Perera O. Martín R. Bentura M. L. Rodrigo J. Lamas S. (1996). Presence of nitric oxide synthase activity in roots and nodules of *Lupinus albus*. *FEBS Lett.* 398 159–164. 10.1016/s0014-5793(96)01232-x 8977098

[B16] De Ron A. M. Sparvoli F. Pueyo J. J. Bazile D. (2017). Editorial: protein crops: food and feed for the future. *Front. Plant Sci.* 8:105. 10.3389/fpls.2017.00105 28220133 PMC5292564

[B17] Dinkelaker B. Hengeler C. Marschner H. (1995). Distribution and function of proteoid roots and other root clusters. *Bot. Acta* 108 183–200. 10.1111/j.1438-8677.1995.tb00850.x

[B18] Dinkelaker B. Römheld V. Marschner H. (1989). Citric acid excretion and precipitation of calcium citrate in the rizosphere of white lupin (*Lupinus albus* L.). *Plant Cell Environ.* 12 285–292. 10.1111/j.1365-3040.1989.tb01942.x

[B19] Doyle A. D. Moore K. J. Herridge D. F. (1989). The narrow-leafed lupin (*Lupinus angustifolius* L.) as a nitrogen-fixing rotation crop for cereal production. III. Residual effects of lupins on subsequent cereal crops. *Aust. J. Agric. Res.* 39 1029–1037. 10.1071/ar9881029

[B20] Egle K. Römer W. Keller H. (2003). Exudation of low molecular weight organic acids by *Lupinus albus* L., *Lupinus angustifolius* L. and *Lupinus luteus* L. as affected by phosphorus supply. *Agronomie* 23 511–518.

[B21] Esfahani M. N. Kusano M. Nguyen K. H. Watanabe Y. Ha C. V. Saito K. (2016). Adaptation of the symbiotic *Mesorhizobium*-chickpea relationship to phosphate deficiency relies on reprogramming of whole-plant metabolism. *Proc. Natl. Acad. Sci. U.S.A.* 113 4610–4619. 10.1073/pnas.1609440113 27450089 PMC4987776

[B22] Fedorova E. Redondo F. J. Koshiba T. Pueyo J. J. de Felipe M. R. Lucas M. M. (2005). Aldehyde oxidase (AO) in the root nodules of *Lupinus albus* and *Medicago truncatula*: identification of AO in meristematic and infection zones. *Mol. Plant-Microbe Interact.* 218 405–413. 10.1094/MPMI-18-0405 15915639

[B23] Fedorova E. E. De Felipe M. R. Pueyo J. J. Lucas M. M. (2007). Conformation of cytoskeletal elements during the division of infected *Lupinus albus* L. nodule cells. *J. Exp. Bot.* 58 2225–2236. 10.1093/jxb/erm083 17525079

[B24] Fernández-Pascual M. Pueyo J. J. de Felipe M. R. Golvano M. P. Lucas M. M. (2007). Singular features of the *Bradyrhizobium-Lupinus* symbiosis. *Dyn. Soil Dyn. Plant* 1 1–16.

[B25] Figueredo M. S. Formey D. Rodríguez J. Ibanez F. Hernandez G. Fabra A. (2020). Identification of miRNAs linked to peanut nodule functional processes. *J. Biosci.* 45:62. 10.1007/s12038-020-00034-5 32385221

[B26] Fischer J. J. Beatty P. H. Allen G. Good A. G. Douglas G. Muench D. G. (2013). Manipulation of microRNA expression to improve nitrogen use efficiency. *Plant Sci.* 210 70–81. 10.1016/j.plantsci.2013.05.009 23849115

[B27] Franco-Zorrilla J. M. Martin A. C. Leyva A. Paz-Ares J. (2005). Interaction between phosphate starvation, sugar, and cytokinin signaling in *Arabidopsis* and the roles of cytokinin receptors CRE1/AHK4 and AHK3. *Plant Physiol.* 138 847–857. 10.1104/pp.105.060517 15923327 PMC1150402

[B28] Fujiishi M. Maejima E. Watanabe T. (2020). Effect of mixed cropping with lupin (*Lupinus albus* L.) on growth and nitrogen uptake in pasture grasses grown under manure application. *Arch. Agron. Soil Sci.* 66 96–109. 10.1080/03650340.2019.1600673

[B29] Gallardo C. Hufnagel B. Casset C. Alcon C. Garcia F. Divol F. (2019). Anatomical and hormonal description of rootlet primordium development along white lupin cluster root. *Physiol. Plant.* 165 4–16. 10.1111/ppl.12714 29493786

[B30] Gardner W. K. Boundy K. A. (1983). The acquisition of phosphorus by *Lupinus albus* L. IV. The effect of interplanting wheat and white lupin on the growth and mineral composition of the two species. *Plant Soil* 70 391–402. 10.1007/bf02374894

[B31] Gardner W. K. Parbery D. G. Barber D. A. (1982). The acquisition of phosphorus by *Lupinus albus* L. I. Some characteristics of the soil/root interface. *Plant Soil* 67 19–32. 10.1007/bf02374724

[B32] Garneau M. G. Tan Q. Tegeder M. (2018). Function of pea amino acid permease AAP6 in nodule nitrogen metabolism and export, and plant nutrition. *J. Exp. Bot.* 69 5205–5219. 10.1093/jxb/ery289 30113690 PMC6184819

[B33] Gilbert G. A. Knight J. D. Vance C. P. Allan D. L. (2000). Proteoid root development of phosphorus deficient lupin is mimicked by auxin and phosphonate. *Ann. Bot.* 85 921–928. 10.1006/anbo.2000.1133

[B34] González-Sama A. Coba de la Peña T. Kevei Z. Mergaert P. Lucas M. M. de Felipe M. R. (2006). Nuclear DNA endoreduplication and expression of the mitotic inhibitor Ccs52 associated to determinate and lupinoid nodule organogenesis. *Mol. Plant-Microbe Interact.* 19 173–180. 10.1094/mpmi-19-0173 16529379

[B35] González-Sama A. Lucas M. M. de Felipe M. R. Pueyo J. J. (2004). An unusual infection mechanism and nodule morphogenesis in white lupin (*Lupinus albus*). *New Phytol.* 163 371–380. 10.1111/j.1469-8137.2004.01121.x 33873628

[B36] Guo R. Q. Silsbury J. H. Graham R. D. (1992). Effect of four nitrogen compounds on nodulation and nitrogen fixation in faba bean, white lupin and medic plants. *Funct. Plant Biol.* 19 501–508. 10.1071/pp9920501

[B37] Güsewell S. (2004). N:P ratios in terrestrial plants: variation and functional significance. *New Phytol.* 164 243–266. 10.1111/j.1469-8137.2004.01192.x 33873556

[B38] Hawkins H. J. Wolf G. Stock W. D. (2005). Cluster roots of *Leucadendron laureolum* (Proteaceae) and *Lupinus albus* (Fabaceae) take up glycine intact: an adaptive strategy to low mineral nitrogen in soils? *Ann. Bot.* 96 1275–1282. 10.1093/aob/mci279 16223736 PMC4247078

[B39] Hernández G. Valdés-López O. Ramírez M. Goffard N. Weiller G. Aparicio-Fabre R. (2009). Global changes in the transcript and metabolic profiles during symbiotic nitrogen fixation in phosphorus-stressed common bean plants. *Plant Physiol.* 151 1221–1238. 10.1104/pp.109.143842 19755543 PMC2773089

[B40] Hocking P. J. Jeffery S. (2004). Cluster-root production and organic anion exudation in a group of old-world lupins and a new-world lupin. *Plant Soil* 258 135–150. 10.1023/b:plso.0000016544.18563.86

[B41] Høgh-Jensen H. Schjoerring J. K. Soussana J.-F. (2002). The influence of phosphorus deficiency on growth and nitrogen fixation of white clover plants. *Ann. Bot.* 90 745–753. 10.1093/aob/mcf260 12451030 PMC4240371

[B42] Horst W. J. Waschkies C. (1987). Phosphorus-nutrition of spring wheat (*Triticum aestivum* L.) in mixed culture with white lupin (*Lupinus albus* L.). *J. Plant Nutr. Soil Sci.* 150 1–8.

[B43] Hu B. Chu C. (2020). Nitrogen–phosphorus interplay: old story with molecular tale. *New Phytol.* 225 1455–1460. 10.1111/nph.16102 31400226

[B44] Hufnagel B. Marques A. Soriano A. Marquès L. Divol F. Doumas P. (2020). High-quality genome sequence of white lupin provides insight into soil exploration and seed quality. *Nat. Comm.* 11:492. 10.1038/s41467-019-14197-9 31980615 PMC6981116

[B45] Ibáez F. Wall L. Fabra A. (2017). Starting points in plant-bacteria nitrogen fixing symbioses: intercellular invasion of the roots. *J. Exp. Bot.* 68 1905–1918. 10.1093/jxb/erw387 27756807

[B46] Jauni M. Ramula S. (2017). Demographic mechanisms of disturbance and plant diversity promoting the establishment of invasive *Lupinus polyphyllus*. *J. Plant Ecol.* 10 510–517.

[B47] Johnson J. F. Allan D. L. Vance C. P. Weiblen G. (1996). Root carbon dioxide fixation by phosphorus-deficient *Lupinus albus*. *Plant Physiol.* 112 19–30. 10.1104/pp.112.1.19 12226371 PMC157919

[B48] Kihara T. Wada T. Suzuki Y. Hara T. Koyama H. (2003). Alteration of citrate metabolism in cluster roots of white lupin. *Plant Cell Physiol.* 44 901–908. 10.1093/pcp/pcg115 14519771

[B49] Kleinert A. Venter M. Kossmann J. Valentine A. (2014). The reallocation of carbon in P deficient lupins affects biological nitrogen fixation. *J. Plant Physiol.* 171 1619–1624. 10.1016/j.jplph.2014.07.017 25155758

[B50] Lambers H. Bishop J. G. Hopper S. D. Laliberté E. Zúiga-Feest A. (2012). Phosphorus-mobilization ecosystem engineering: the roles of cluster roots and carboxylate exudation in young P-limited ecosystems. *Ann. Bot.* 110 329–348. 10.1093/aob/mcs130 22700940 PMC3394658

[B51] Lambers H. Clements J. C. Nelson M. N. (2013). How a phosphorus-acquisition strategy based on carboxylate exudation powers the success and agronomic potential of lupine(*Lupinus*, *Fabaceae*). *Am. J. Bot.* 100 263–288. 10.3732/ajb.1200474 23347972

[B52] Lamont B. (2003). Structure, ecology and physiology of root clusters – a review. *Plant Soil* 248 1–19. 10.1023/a:1022314613217

[B53] Le Roux M. R. Ward C. L. Botha F. C. Valentine A. J. (2006). Routes of pyruvate synthesis in phosphorus-deficient lupin roots and nodules. *New Phytol.* 169 399–408. 10.1111/j.1469-8137.2005.01594.x 16411942

[B54] Li H. G. Shen J. B. Zhang F. S. Tang C. X. Lambers H. (2008). Is there a critical level of shoot phosphorus concentration for cluster-root formation in *Lupinus albus*? *Funct. Plant Biol.* 35 328–336. 10.1071/FP07222 32688788

[B55] Liese R. Schulze J. Cabeza R. A. (2017). Nitrate application or P deficiency induce a decline in *Medicago truncatula* N_2_-fixation by similar changes in the nodule transcriptome. *Sci. Rep.* 7:46264. 10.1038/srep46264 28393902 PMC5385875

[B56] Lin J.-S. Li X. Luo Z. Mysore K. S. Wen J. Xie F. (2018). NIN interacts with NLPs to mediate nitrate inhibition of nodulation in *Medicago truncatula*. *Nat. Plants* 44 942–952. 10.1038/s41477-018-0261-3 30297831

[B57] Liu J. Samac D. A. Bucciarelli B. Allan D. L. Vance C. P. (2005). Signaling of phosphorus deficiency-induced gene expression in white lupin requires sugar and phloem transport. *Plant J.* 41 257–268. 10.1111/j.1365-313x.2004.02289.x 15634202

[B58] Liu J. Uhde-Stone C. Li A. Vance C. Allan D. (2001). A phosphate transporter with enhanced expression in proteoid roots of white lupin (*Lupinus albus* L.). *Plant Soil* 237 257–266. 10.1023/A:1013396825577

[B59] Lucas M. M. Stoddard F. L. Annicchiarico P. Frías J. Martínez-Villaluenga C. Sussmann D. (2015). The future of lupin as a protein crop in Europe. *Front. Plant Sci.* 6:705. 10.3389/fpls.2015.00705 26442020 PMC4561814

[B60] Lucena C. Porras R. García M. J. Alcántara E. Pérez-Vicente R. Zamarreo A. M. (2019). Ethylene and phloem signals are involved in the regulation of responses to Fe and P deficiencies in roots of strategy I plants. *Front. Plant Sci.* 10:1237. 10.3389/fpls.2019.01237 31649701 PMC6795750

[B61] Lynch J. P. Ho M. D. (2005). Rhizoeconomics: carbon costs of phosphorus acquisition. *Plant Soil* 269 45–56. 10.1007/s11104-004-1096-4

[B62] Martín A. C. del Pozo J. C. Iglesias J. Rubio V. Solano R. de La Pea A. (2000). Influence of cytokinins on the expression of phosphate starvation responsive genes in *Arabidopsis*. *Plant J.* 24 559–567. 10.1046/j.1365-313x.2000.00893.x 11123795

[B63] McCluskey J. Herdman L. Skene K. R. (2004). Iron deficiency induces changes in metabolism of citrate in lateral roots and cluster roots of *Lupinus albus*. *Physiol. Plant.* 121 586–594. 10.1111/j.1399-3054.2004.00372.x

[B64] Menge D. N. L. Hedin L. O. Pacala S. W. (2012). Nitrogen and phosphorus limitation over long-term ecosystem development in terrestrial ecosystems. *PLoS One* 7:e42045. 10.1371/journal.pone.0042045 22870281 PMC3411694

[B65] Mortimer P. E. Pérez-Fernández M. A. Valentine A. J. (2008). The role of arbuscular mycorrhizal colonization in the carbon and nutrient economy of the tripartite symbiosis with nodulated *Phaseolus vulgaris*. *Soil Biol. Biochem.* 40 1019–1027. 10.1016/j.soilbio.2007.11.014

[B66] Müller J. Gödde V. Niehaus K. Zörb C. (2015). Metabolic adaptations of white lupin roots and shoots under phosphorus deficiency. *Front. Plant Sci.* 6:1014. 10.3389/fpls.2015.01014 26635840 PMC4656794

[B67] Nagarajan V. K. Smith A. P. (2012). Ethylene’s role in phosphate starvation signaling: more than just a root growth regulator. *Plant Cell Physiol.* 53 277–286. 10.1093/pcp/pcr186 22199374

[B68] Nascimento F. X. Tavares M. J. Rossi M. J. Glick B. R. (2018). The modulation of leguminous plant ethylene levels by symbiotic rhizobia played a role in the evolution of the nodulation process. *Heliyon* 4:e01068. 10.1016/j.heliyon.2018.e01068 30603701 PMC6304460

[B69] Neumann G. Martinoia E. (2002). Cluster roots – an underground adaptation for survival in extreme environments. *Trends Plant Sci.* 7 162–167. 10.1016/s1360-1385(02)02241-0 11950612

[B70] O’Rourke J. A. Yang S. S. Miller S. S. Bucciarelli B. Liu J. Rydeen A. (2013). An RNA-Seq transcriptome analysis of orthophosphate-deficient white lupin reveals novel insights into phosphorus acclimation in plants. *Plant Physiol.* 161 705–724. 10.1104/pp.112.209254 23197803 PMC3561014

[B71] Paungfoo-Lonhienne C. Lonhienne T. G. Rentsch D. Robinson N. Christie M. Webb R. I. (2008). Plants can use protein as a nitrogen source without assistance from other organisms. *Proc. Natl. Acad. Sci. USA* 105 4524–4529. 10.1073/pnas.0712078105 18334638 PMC2393761

[B72] Paungfoo-Lonhienne C. Schenk P. M. Lonhienne T. G. A. Brackin R. Meier S. Rentsch D. (2009). Nitrogen affects cluster root formation and expression of putative peptide transporters. *J. Exp. Bot.* 60 2665–2676. 10.1093/jxb/erp111 19380419 PMC2692012

[B73] Pearse S. J. Veneklaas E. J. Cawthray G. R. Bolland M. D. A. Lambers H. (2006). Carboxylate release of wheat, canola and 11 grain legume species as affected by phosphorus status. *Plant Soil* 288 127–139. 10.1007/s11104-006-9099-y

[B74] Quiñones M. A. Ruiz-Díez B. Fajardo S. López-Berdonces M. A. Higueras P. L. Fernández-Pascual M. (2013). *Lupinus albus* plants acquire mercury tolerance when inoculated with and Hg-resistant *Bradyrhizobium* strain. *Plant Physiol. Biochem.* 73 168–175. 10.1016/j.plaphy.2013.09.015 24125840

[B75] Rath M. Salas J. Parhy B. Norton R. Menakuru H. Sommerhalter M. (2010). Identification of genes induced in proteoid roots of white lupin under nitrogen and phosphorus deprivation, with functional characterization of a formamidase. *Plant Soil* 334 137–150. 10.1007/s11104-010-0373-7

[B76] Raven J. A. (2012). Protein turnover and plant RNA and phosphorus requirements in relation to nitrogen fixation. *Plant Sci.* 188–189. 10.1016/j.plantsci.2012.02.010 22525241

[B77] Razavi B. S. Hoang D. T. T. Blagodatskaya E. Kuzyakov Y. (2017). Mapping the footprint of nematodes in the rhizosphere: cluster root formation and spatial distribution of enzyme activities. *Soil Biol. Biochem.* 115 213–220. 10.1016/j.soilbio.2017.08.027

[B78] Rosendahl L. Vance C. P. Pedersen W. B. (1990). Products of dark CO_2_ fixation in pea root nodules support bacteroid metabolism. *Plant Physiol.* 93 12–19. 10.1104/pp.93.1.12 16667422 PMC1062460

[B79] Sa T.-M. Israel D. W. (1991). Energy status and functioning of phosphorus-deficient soybean nodules. *Plant Physiol.* 97 928–935. 10.1104/pp.97.3.928 16668533 PMC1081106

[B80] Sas L. Rengel Z. Tang C. (2002). The effect of nitrogen nutrition on cluster root formation and proton extrusion by *Lupinus albus*. *Ann. Bot.* 89 435–442. 10.1093/aob/mcf066 12096804 PMC4233878

[B81] Schmidt S. Mason M. Sangtiean T. Stewart G. (2003). Do cluster roots of *Hakea actities* (*Proteaceae*) acquire complex organic nitrogen? *Plant Soil* 248 157–165. 10.1023/A:1022352415728

[B82] Schmidt S. Stewart G. R. (1999). Glycine metabolism by plant roots and its occurrence in Australian plant communities. *Funct. Plant Biol.* 26 253–264. 10.1071/pp98116

[B83] Schultze M. Kondorosi A. (1998). Regulation of symbiotic root nodule development. *Annu. Rev. Genet.* 32 33–57. 10.1146/annurev.genet.32.1.33 9928474

[B84] Schulze J. Temple G. Temple S. J. Beschow H. Vance C. P. (2006). Nitrogen fixation by white lupin under phosphorus deficiency. *Ann. Bot.* 98 731–740. 10.1093/aob/mcl154 16855013 PMC2806177

[B85] Secco D. Shou H. Whelan J. Berkowitz O. (2014). RNA-seq analysis identifies an intricate regulatory network controlling cluster root development in white lupin. *BMC Genomics* 15:230. 10.1186/1471-2164-15-230 24666749 PMC4028058

[B86] Skene K. R. (2000). Pattern formation in cluster roots: some developmental and evolutionary considerations. *Ann. Bot.* 85 901–908. 10.1006/anbo.2000.1140

[B87] Skene K. R. James W. M. (2000). A comparison of the effects of auxin on cluster root initiation and development in *Grevillea robusta* Cunn. ex R. Br. (Proteaceae) and in the genus *Lupinus* (Leguminosae). *Plant Soil* 219 221–229.

[B88] Sulieman S. Tran L.-S. P. (2015). Phosphorus homeostasis in legume nodules as an adaptive strategy to phosphorus deficiency. *Plant Sci.* 239 36–43. 10.1016/j.plantsci.2015.06.018 26398789

[B89] Tegeder M. (2014). Transporters involved in source to sink partitioning of amino acids and ureides: opportunities for crop improvement. *J. Exp. Bot.* 65 1865–1878. 10.1093/jxb/eru012 24489071

[B90] Thuynsma R. Valentine A. Kleinert A. (2014a). Phosphorus deficiency affects the allocation of below-ground resources to combined cluster roots and nodules in *Lupinus albus*. *J. Plant Physiol.* 171 285–291. 10.1016/j.jplph.2013.09.001 24129121

[B91] Thuynsma R. Valentine A. Kleinert A. (2014b). Short-term supply of elevated phosphate alters the belowground carbon allocation costs and functions of lupin cluster roots and nodules. *J. Plant Physiol.* 171 648–654. 10.1016/j.jplph.2014.01.003 24709158

[B92] Tomasi N. Kreitzschmar T. Espen L. Weisskopf L. Fuglsang A. T. Palmgren M. G. (2009). Plasma membrane H+-ATPase-dependent citrate exudation from cluster roots of phosphate-deficient white lupin. *Plant Cell Environ.* 32 465–475. 10.1111/j.1365-3040.2009.01938.x 19183296

[B93] Tomasi N. Weisskopf L. Renella G. Landi G. Pinton R. Varanini Z. (2008). Flavonoids of white lupin roots participate in phosphorus mobilization from soil. *Soil Biol. Biochem.* 40 1971–1974. 10.1016/j.soilbio.2008.02.017

[B94] Trinick M. J. (1977). Vesicular-arbuscular infection and soil phosphorus utilization in *Lupinus* spp. *New Phytol.* 78 297–304. 10.1111/j.1469-8137.1977.tb04833.x

[B95] Udvardi M. Poole P. S. (2013). Transport and metabolism in legume-rhizobia symbioses. *Annu. Rev. Plant Biol.* 64 781–805. 10.1146/annurev-arplant050312-120235 23451778

[B96] Ueda Y. Yanagisawa S. (2019). Perception, transduction, and integration of nitrogen and phosphorus nutritional signals in the transcriptional regulatory network in plants. *J. Exp. Bot.* 70 3709–3717. 10.1093/jxb/erz148 30949701

[B97] Uhde-Stone C. Gilbert G. Johnson J. M.-F. Litjens R. Zinn K. E. Temple S. J. (2003). Acclimation of white lupin to phosphorus deficiency involves enhanced expression of genes related to organic acid metabolism. *Plant Soil* 248 99–116. 10.1007/978-94-010-0243-1_8

[B98] Valentine A. J. Benedito V. A. Kang Y. (2011). “Legume nitrogen fixation and soil abscisic stress: from physiology to genomics and beyond,” in *Nitrogen Metabolism in Plants in the Post-Genomic Era*, eds Foyer C. H. Zhang H. (Hoboken, NJ: Wiley Blackwell), 207–248. 10.1002/9781444328608.ch9

[B99] Valentine A. J. Kleinert A. Benedito V. A. (2017). Adaptive strategies for nitrogen metabolism in phosphate deficient legume nodules. *Plant Sci.* 256 46–52. 10.1016/j.plantsci.2016.12.010 28167037

[B100] Vance C. P. (2001). Symbiotic nitrogen fixation and phosphorus acquisition. Plant nutrition in a world of declining renewable resources. *Plant Physiol.* 127 390–397. 10.1104/pp.127.2.390 11598215 PMC1540145

[B101] Vance C. P. Uhde-Stone C. Allan D. L. (2003). Phosphorus acquisition and use: critical adaptations by plants for securing a nonrenewable resource. *New Phytol.* 157 423–447. 10.1046/j.1469-8137.2003.00695.x 33873400

[B102] Vardien W. Steenkamp E. T. Valentine A. J. (2016). Legume nodules from nutrient-poor soils exhibit high plasticity of cellular phosphorus recycling and conservation during variable phosphorus supply. *J Plant Physiol.* 191 73–81. 10.1016/j.jplph.2015.12.002 26720212

[B103] Venuti S. Zanin L. Marroni F. Franco A. Morgante M. Pinton R. (2019). Physiological and transcriptomic data highlight common features between iron and phosphorus acquisition mechanisms in white lupin roots. *Plant Sci.* 285 110–121. 10.1016/j.plantsci.2019.04.026 31203875

[B104] Vitousek P. M. Porder S. Houlton B. Z. Chadwick O. A. (2010). Terrestrial phosphorus limitation: mechanisms, implications, and nitrogen-phosphorus interactions. *Ecol. Appl.* 20 5–15. 10.1890/08-0127.1 20349827

[B105] Wang B. L. Tang X. Y. Cheng L. Y. Zhang A. Z. Zhang W. H. Zhang F. S. (2010). Nitric oxide is involved in phosphorus deficiency-induced cluster-root development and citrate exudation in white lupin. *New Phytol.* 187 1112–1123. 10.1111/j.1469-8137.2010.03323.x 20553395

[B106] Wang X. Ding W. Lambers H. (2019a). Nodulation promotes cluster-root formation in *Lupinus albus* under low phosphorus conditions. *Plant Soil* 439 233–242. 10.1007/s11104-018-3638-1

[B107] Wang Y. Yang W. Zuo Y. Zhu L. Hastwell A. H. Chen L. (2019b). GmYUC2a mediates auxin biosynthesis during root development and nodulation in soybean. *J. Exp. Bot.* 70 3165–3176. 10.1093/jxb/erz144 30958883 PMC6598056

[B108] Wang Z. Straub D. Yang H. Kania A. Shen J. Ludewig U. (2014). The regulatory network of cluster-root function and development in phosphate-deficient white lupin (*Lupinus albus*) identified by transcriptome sequencing. *Physiol. Plant.* 151 323–338. 10.1111/ppl.12187 24635386

[B109] Wang Z. Rahman A. B. M. M. Wang G. Ludewig U. Shen J. Neumann G. (2015). Hormonal interactions during cluster-root development in phosphate-deficient white lupin (*Lupinus albus* L.). *J. Plant. Physiol.* 177 74–82. 10.1016/j.jplph.2014.10.022 25668414

[B110] Watanabe T. Okamoto M. Misawa S. Urayama M. Osaki M. (2006). Different characteristics of nitrogen utilization between lupin and soybean: can lupin utilize organic nitrogen in soils? *Can. J. Bot.* 84 20–27. 10.1139/b05-136

[B111] Watt M. Evans J. R. (1999). Proteoid roots. Physiology and development. *Plant Physiol.* 121 317–323. 10.1104/pp.121.2.317 10517822 PMC1539228

[B112] Welch R. M. Graham R. D. (2004). Breeding for micronutrients in staple food crops from a human nutrition perspective. *J. Exp. Bot.* 55 353–364. 10.1093/jxb/erh064 14739261

[B113] Xu H. Li Y. Zhang K. Li M. Fu S. Tian Y. (2020). miR169c-NFYA-C-ENOD40 modulates nitrogen inhibitory effects in soybean nodulation. *New Phytol*. 10.1111/nph.17115 Online ahead of print 33245793

[B114] Zhang W.-H. Ryan P. R. Tyerman S. D. (2004). Citrate-permeable channels in the plasma membrane of cluster roots from white lupin. *Plant Physiol.* 136 3771–3783. 10.1104/pp.104.046201 15516510 PMC527174

[B115] Zhou Y. Neuhäuser B. Neumann G. Ludewig U. (2019). LaALMT1 mediates malate release from phosphorus-deficient white lupin root tips and metal root to shoot translocation. *Plant Cell Environ.* 43 1691–1706. 10.1111/pce.13762 32239684

[B116] Zhu Y. Y. Zeng H. Q. Dong C. X. Yin X. M. Shen Q. R. Yang Z. M. (2010). microRNA expression profiles associated with phosphorus deficiency in white lupin (*Lupinus albus* L.). *Plant Sci.* 178 23–29. 10.1016/j.plantsci.2009.09.011

